# Intraoperative relaxed muscle positioning technique results in a tertiary Center for Thyroid Orbitopathy Related Strabismus

**DOI:** 10.1186/s12886-018-0974-0

**Published:** 2018-11-23

**Authors:** Ahmet Murat Sarici, Burak Mergen, Velittin Oguz, Cezmi Dogan

**Affiliations:** Department of Ophthalmology, Cerrahpasa Medical Faculty, Istanbul University, Cerrahpasa School of Medicine, 34098, Fatih, Istanbul, Turkey

**Keywords:** Thyroid orbitopathy, Intraoperative relaxed muscle positioning, Restrictive strabismus

## Abstract

**Background:**

Previous techniques resulted with low rates of successful fusion after the surgeries and high necessity of additional surgeries in the treatment of thyroid orbitopathy related strabismus. In this study, reviewing the results of our patients who had surgical correction with relaxed muscle positioning technique due to thyroid orbitopathy related strabismus to evaluate the effectiveness of the surgery was aimed.

**Methods:**

The medical records of 8 patients who had surgical correction with intraoperative relaxed muscle positioning (IRMP) technique were studied retrospectively. The extent of strabismus was determined with prism cover test both at near and distance. The extent of recession was determined by marking the natural place of the released tendon during the primary position. The muscle then sutured to the globe at this precise point.

**Results:**

Seven eyes (87.5%) of 8 patients had orthophoria after the surgery and they reported no diplopia in primary and reading gaze. The mean age of the patients was 51 ± 8.8 years. The mean follow-up time was 32.7 ± 18.5 months. Three patients had inferior rectus recession (IRR), 3 had bilateral medial rectus recession (MRR), 1 had only right MRR and 1 had combined MRR with IRR during the surgical correction of the strabismus. The mean amount of recession for IR was 7.5 ± 1.34 mm and it was 6.75 ± 0.95 mm for the MR muscles. The mean prism diopter before the surgery was 37.8 ± 23.3 and it was 0 after surgery except only one of the patients who had > 60 prism diopter (PD) left esotropia (ET) before surgery and had 30 PD left ET after surgery (3.3 ± 9.4).

**Conclusion:**

IRMP technique is a unique option for the surgical correction of thyroid orbitopathy related strabismus. By showing a dramatic increase in the quality of life of the patients, our surgical results are promising despite limited number of patients.

**Electronic supplementary material:**

The online version of this article (10.1186/s12886-018-0974-0) contains supplementary material, which is available to authorized users.

## Introduction

Thyroid eye disease (TED) is the most common extra-thyroid finding of Graves’ disease [1]. Restrictive myopathy is a common manifestation of Graves’ disease causing daily life disturbing diplopia [2]. Inferior and medial rectus muscles are the most commonly involved muscles. Active cigarette smoking is the only reported predictor linked to increased rates of strabismus surgery [3].

Although new approaches such as botulinum toxin injection were introduced, the main surgical technique for correcting the TED associated strabismus has been muscle recessions with or without adjustable sutures for years. However, low rates of successful fusion after the surgeries (47–92% with adjustable suture [4–6] and 38–82% without adjustable suture [6, 7]) and high necessity of additional surgeries with these techniques created a need for a better surgical approach (reoperation rates were 5–17% vs. 18–40% respectively [6, 8, 9]).

After Prendiville et al. [10] emphasized the importance of the surgery tailored to address restriction of ductions to improve the success of the surgery; Nguyen et al. [11] confirmed the impact of this conclusion in a large series of patients with Graves’ ophthalmopathy. They found an improved success with surgery aimed at correcting the restriction of duction rather than correction of deviation. Later in another report, a new surgical technique was introduced which the authors called *“intraoperative relaxed muscle positioning technique”* where they determined the fixation point for muscle recession intraoperatively after disinsertion by resting the relaxed muscle on the globe in the primary position. 87.5% of their patients showed no diplopia in primary and reading position with a very low reoperation rate (8%) [12]. The result of the study was encouraging, and we started to apply this technique on our patients with TED associated strabismus.

Here we report the surgical outcome of a short series of patients with TED associated strabismus who had surgery with intraoperative relaxed muscle positioning technique and their change in terms of the quality of life (QoL).

## Methods

The medical records of 8 patients who had surgical correction with intraoperative relaxed muscle positioning technique between May 2013 and June 2016 were studied retrospectively. The following data was recorded for all patients: age, sex, history of smoking, thyroid status without treatment (hyperthyroid, hypothyroid, euthyroid), duration of thyroid disease, duration of diplopia or strabismus, previous systemic treatments for thyroid disease (methimazole, levothyroxine, steroid, selenium, orbital radiotherapy, radioactive iodine, and thyroidectomy), and history of orbital decompression surgery. All patients were questioned about diplopia in primary position and downgaze. The extent of strabismus was determined with prism cover test both at near and distance. Excellent result for the surgery was defined as no diplopia in primary and reading position. Approval from the local ethical committee was received. The tenets of the Declaration of Helsinki were followed.

The diagnosis of TED associated strabismus was made by history of thyroid disease, typical clinical signs (such as lid retraction and proptosis), positive forced duction testing, enlarged extraocular muscles in orbital computed tomography scan, positive thyroid autoantibodies (TSI and anti-TPO), and abnormal thyroid function tests.

**Fig. 1:**
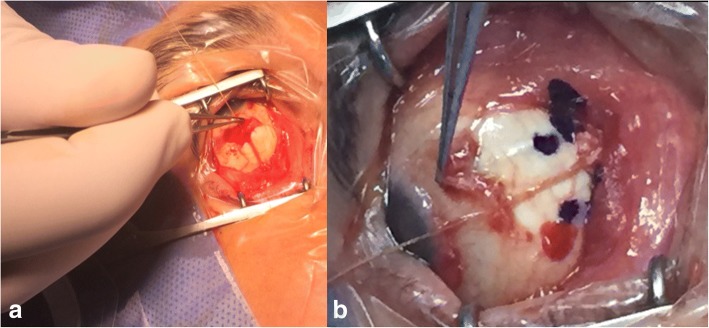
Intraoperative relaxed muscle positioning of the inferior (**a**) and medial (**b**) rectus muscle . After releasing the tendon from its insertion, the precise point is marked for the optimal effect. Then the muscle is sutured onto this precise point

All surgeries were performed by one of the authors (AMS) under general anesthesia. Forced duction testing was done intraoperatively and the extent of recession was determined by marking the natural place of the released tendon during the primary position. The muscle was then sutured to the globe at this precise point (Fig. 1).

For the analysis of the effect of the surgery on the patients with TED associated strabismus, three parameters were determined. Firstly, as described by dal Canto et al. [12], the results were categorized into three variables: (1) excellent success: no diplopia in primary and reading gazes without prisms, (2) good outcome: no diplopia in primary and reading gazes with the use of < 10 prism diopters, and (3) poor outcome: persistent diplopia in primary or reading gazes despite prisms, or the inability of the patient to tolerate the necessary prisms. Secondly, a disease-specific questionnaire developed by Terwee et al. analyzing the QoL for the patients with Graves Ophthalmopathy which in turn produces Graves Ophthalmopathy-QoL (GO-QoL) scores was performed for all patients before surgery and at their last visit. This test includes 8 questions to assess the visual functioning of the patient and another 8 questions assessing his appearance. For all questions, 1–3 points were noted according to the answers. The scores were then calculated as percentages for both visual functioning and appearance by the following formula: total score = ((raw score - 8)/16 × 100). Thirdly the extent of strabismus was evaluated in prism diopters.

Statistical analysis was performed using SPSS version 20.0 (SPSS Inc., Chicago, IL). To compare the paired samples, Wilcoxon signed-rank test was utilized. *p* values below 0.05 were accepted as statistically significant.

## Results

**Fig. 2:**
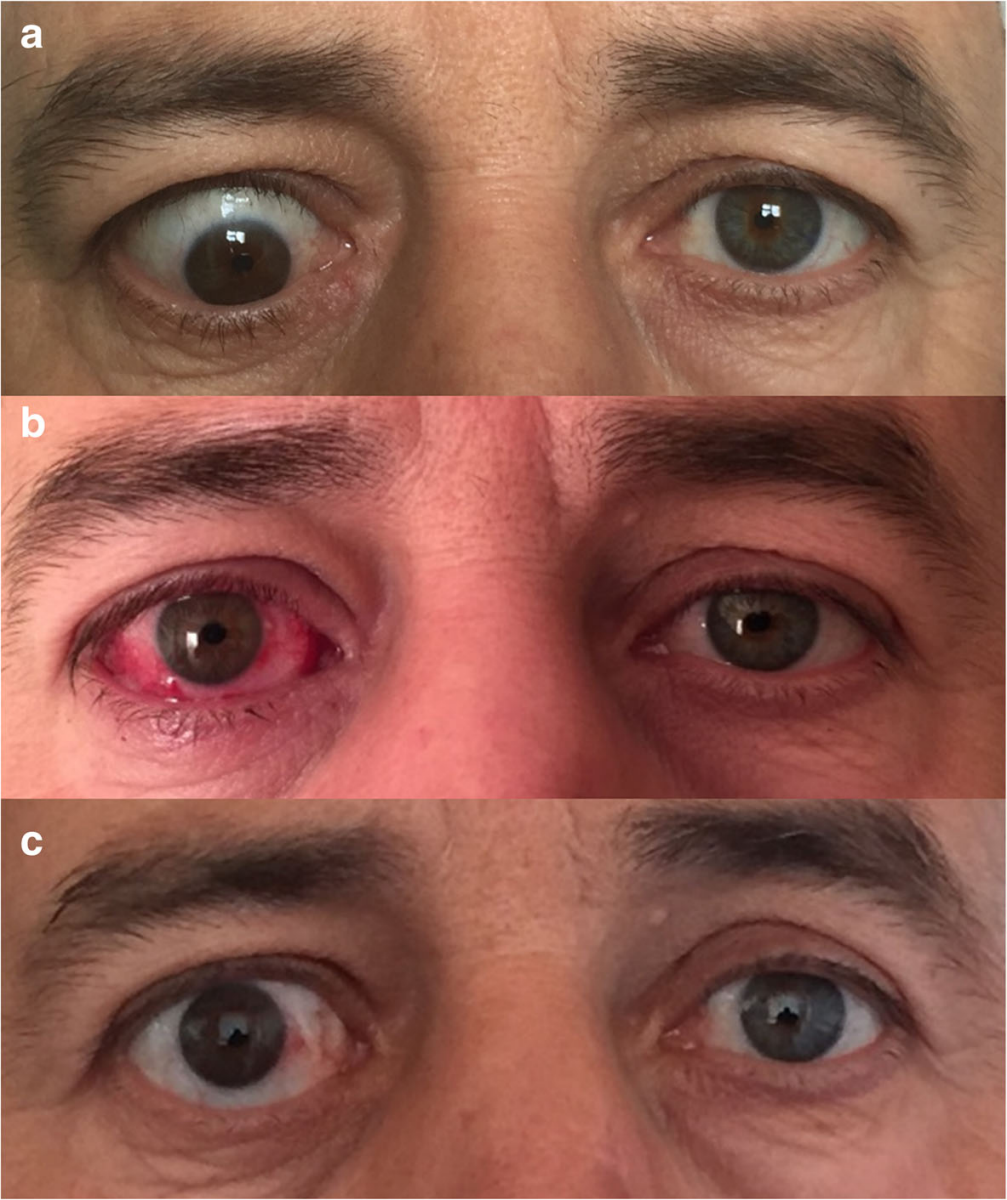
Preoperative (**a**), early postoperative (**b**) and late postoperative (**c**) photos of a patient for whom medial and inferior rectus muscle recession was performed with the intraoperative relaxed muscle positioning technique

**Fig. 3:**
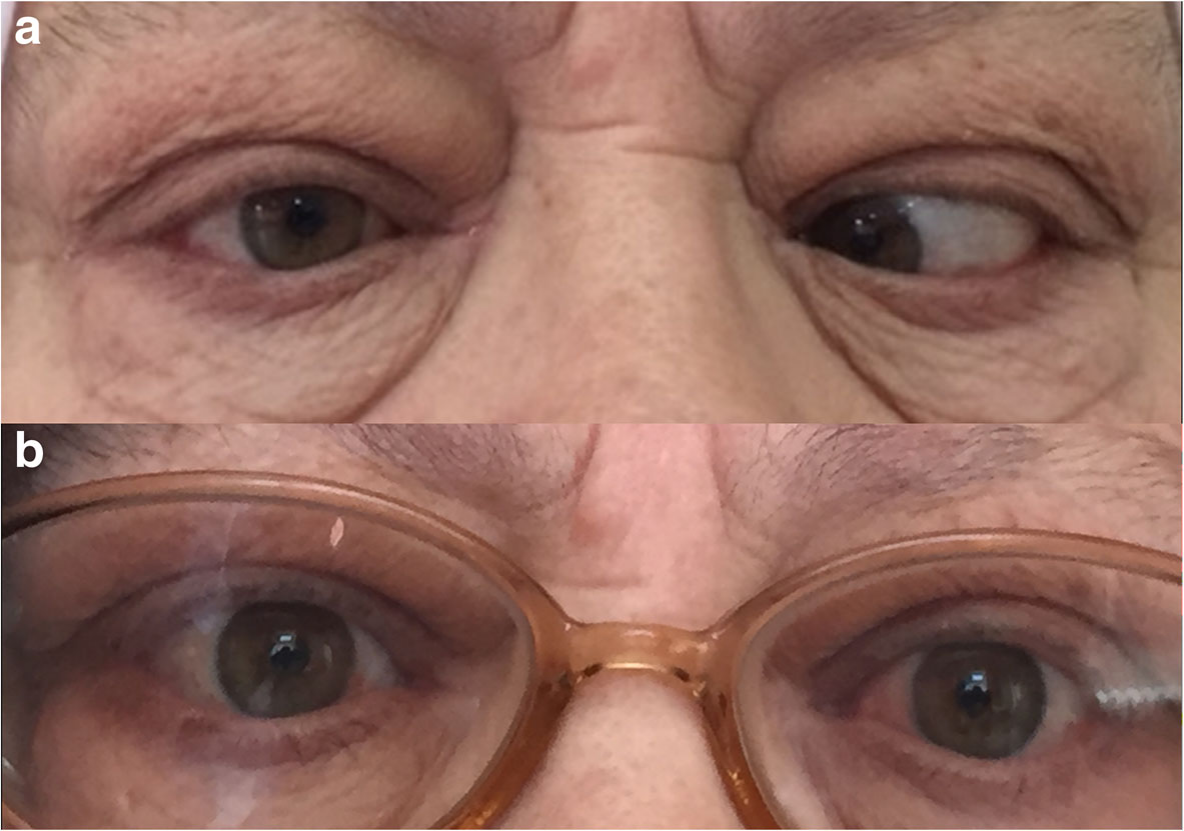
Preoperative photo of a patient with horizontal strabismus (**a**). Late postoperative photo after bilateral MRR surgery (**b**)

Seven eyes (87.5%) of 8 patients had orthophoria after the surgery and they reported no diplopia in the primary and the reading position (excellent success). The mean age of the patients was 51 ± 8.8. The mean follow-up time was 32.7 ± 18.5 months. Male:Female ratio was 5:3 among patients. Three patients had inferior rectus recession (IRR), 3 had bilateral medial rectus recession (MRR), 1 had only right MRR and 1 had combined MRR with IRR during the surgical correction of the strabismus (Figs. 2 and 3). The mean amount of recession for IR was 7.5 ± 1.34 mm and it was 6.75 ± 0.95 mm for the MR muscles. The mean prism diopter before the surgery was 37.3 ± 23.3 and it was 0 after surgery except only one of the patients who had > 60 prism diopter (PD) left esotropia (ET) before surgery and had 30 PD left ET after surgery (3.3 ± 9.4). 7 patients (87.5%) showed excellent success and 1 patient (12.5%) showed poor outcome. The results of the patients are summarized in the Table 1.

**Table 1:** Summary of the clinical findings of the patients

	Age	Preop Strabismus	Surgery	Recession (mm)	Preop PD	Postop PD
Patient #1	49	Right hypotropia	Right IRR	5	10	0
Patient #2	58	Right esotropia, hypotropia	Right MRR + IRR	6,5 + 7,5	60 and 30	0
Patient #3	61	Right esotropia	Bilateral MRR	8 + 8	> 60	30
Patient #4	35	Left esotropia	Bilateral MRR	6,5 + 6	30	0
Patient #5	49	Right exotropia, hypotropia	Right IRR	8	30	0
Patient #6	61	Right esotropia	Right MRR	5,5	30	0
Patient #7	46	Right hypotropia	Right IRR	9	30	0
Patient #8	49	Left hypotropia	Left IRR	8	30	0

IRR: Inferior rectus recession MRR: Medial rectus recession PD: Prism diopter

When GO-QoL questionnaire was performed on the patients, the mean score of visual functioning was 3.1 ± 3.3% and mean score of appearance was 1.6 ± 2.9% before the surgery. However, after surgery at their last visit the mean visual functioning score was found to be 92.2 ± 22.1% and mean appearance score was 89.8 ± 21.4% (*p* < 0.0001) (Additional File 1).

2 patients needed eyelid surgery after strabismus surgery and 1 patient had history of orbital decompression before the strabismus surgery. 2 patients (25%) showed an abnormal head position before surgery that was corrected after surgery. 2 patients (25%) had radioactive iodide treatment, 6 patients (75%) had external beam radiotherapy and 6 patients (75%) had steroid treatment.

## Discussion

TED associated strabismus may cause life disturbing diplopia by restricting the movement of the extraocular muscles. Diplopia occurs in up to 45% of patients with Graves’ orbitopathy [13]. Various surgical techniques such as recessions with or without adjustable sutures or botulinum toxin injection for correcting the thyroid related strabismus were described with differing outcomes [4–9, 14]. Fibrotic and thickened extraocular muscles may complicate the predictability of the surgical results. Thus, a better approach was necessary for estimating the best correction. By addressing this issue, the most successful results were reported with the intraoperative relaxed muscle positioning technique in which the surgeon can determine the extent of the recession intraoperatively.

In the present report, 7 (87.5%) of 8 patients were treated successfully with the intraoperative relaxed muscle positioning technique. First study by Dal Canto et al. [12] utilized this technique on 24 patients with thyroid related strabismus. They reported excellent final outcome in 87.5% of the patients and a clinically acceptable final outcome in all of the patients. However, their results were lacking supportive new studies. Our findings were consistent with their report. One disadvantage of this technique might be dealing with the involvement and restriction of multiple muscles. Because, selecting the proper muscle to recess during surgery might be difficult in case of multiple muscle involvement. However, in one of our patients combined recession of IR with MR resulted with orthotropia.

For the adjustable suture technique, although successful results have been reported, late over-correction problem was reported after recessing the inferior rectus muscle [15, 16]. However a more recent study showed no significant difference between fixed recession and adjustable sutures [17]. Using non-absorbable sutures to decrease the incidence of late over-correction was suggested by some authors, but these sutures have a higher risk of infection. Another approach may be recessing the inferior rectus using a fixed suture on one eye while using adjustable suture on the other eye. This approach may have the advantage of decreasing the amount of recession [18]. Unfortunately, there are no published randomized controlled trials comparing the use of adjustable versus fixed sutures in the TED associated strabismus patients.

Necessity of the second strabismus surgery is another problem for the TED patients. Need for second surgery was reported to be high in the patients with history of decompression surgery [19]. Adjustable suture was designed to address this issue; however, their success rate was reported lower than the intraoperative relaxed muscle positioning technique. Even though muscle resection was not a preferred method in the TED patients, Weldy et al. [20] utilized lateral rectus muscle resection to correct the residual esotropia after a maximal recession of the medial rectus muscle. They reported 91% success rate in 11 patients with large esodeviation.

Another issue regarding the strabismus surgery of the TED patients is choosing the right approach for the patients with a large angle of deviation. In their study, Jellema et al. compared the effect of recessing only the unilateral inferior rectus against recessing also the contralateral superior rectus muscle. Interestingly, less decrease of depression and lower dose-effect response was found in their combined recession group, despite the higher amount of muscle recession [21]. In our study, only unilateral recession of the inferior rectus was performed since the angle of deviation was not large enough. In another study from the same group, Jellema et al. compared the effect of unilateral vs. bilateral medial rectus recession. They found lower dose-effect response in the bilateral medial rectus recession group [22]. This result suggests that bilateral medial rectus recession should be preferred in the patients with large angle deviations.

The most conflicting point in the studies regarding the effect of strabismus surgery in the patients with TED associated strabismus has been the success criteria of the surgery. Most of the studies defined success as no-diplopia in primary and reading position and used a graded scale such as excellent, good, acceptable and failure [4, 12, 23, 24]. Three studies have described a tool to quantify the binocular single vision free of diplopia [25–27] in which they utilized Goldmann perimeter or the Harmswand. However, their necessity for the evaluation of the outcome is questionable, because for the daily life of the patients, the change in their subjective complaints after the surgery are the most important results. Thus, rather than quantifying the binocular single vision free of diplopia with specific instruments, a disease specific questionnaire to evaluate the QoL of the patients has been developed by Terwee et al. Thus, in our study, we adopted a subject-based approach in which a simple graded system (excellent, good, and poor outcome) together with the GO-QoL questionnaire (before surgery and at the last visit) was used for the evaluation of the surgical outcome. Rather than utilization of complicated instruments, this simple approach can be the most efficient subjective success criteria that can be used in any center. In our study, a dramatic increase in the QoL of the patients was observed and 87.5% of the patients had excellent outcome.

**Fig. 4:**
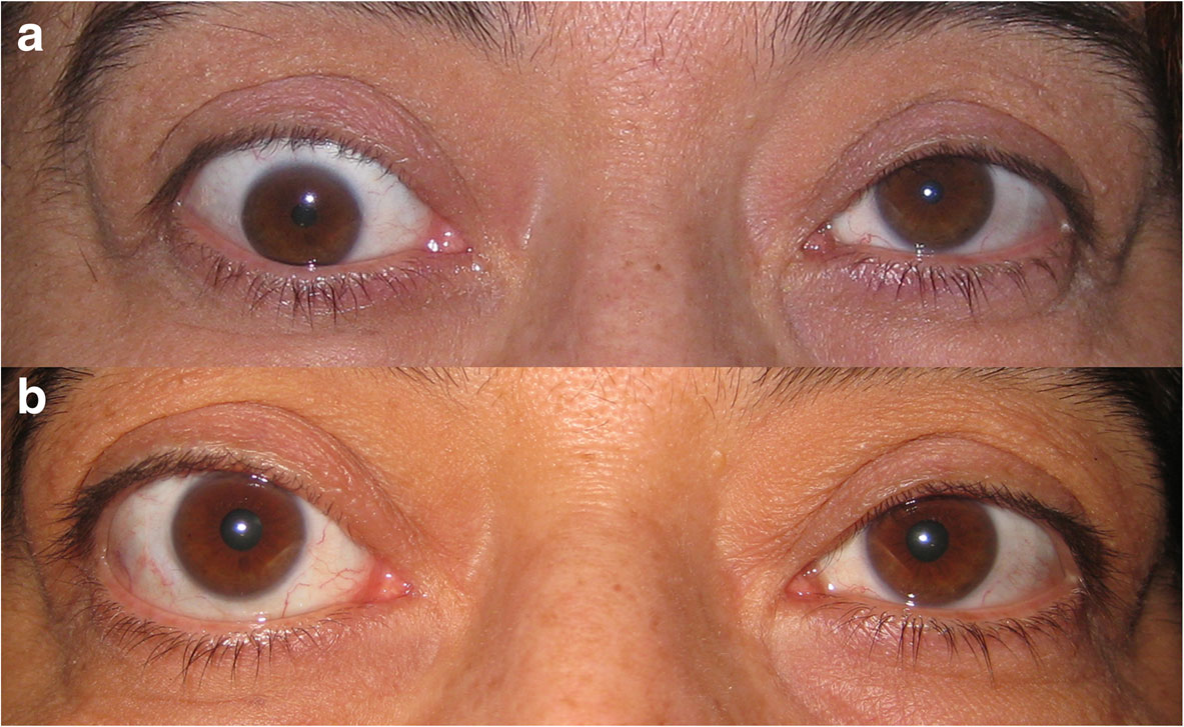
Preoperative (**a**) and postoperative (**b**) photos of a patient with vertical strabismus for whom IRR was performed

The main points to consider during the intraoperative relaxed muscle positioning technique should be the consideration of the fibrotic nature of the rectus muscles during surgery to avoid slippage. Secondly, since the muscles are extremely tight, their positioning for suturing onto the sclera might be challenging. Lower lid retraction can develop after the inferior rectus recessions because of its relationship with the lower lid retractors (Fig. 4). Further surgeries to correct the lid retraction can be considered after the stabilization. Some modifications during the muscle positioning can be developed in future.

In conclusion, in this study we suggested that the use of intraoperative relaxed muscle positioning technique might be a very successful option for the correction of the TED associated strabismus and this technique might provide a dramatic increase in the QoL of the patients. Low sample size and absence of a control group with a different surgical technique were the limitations of our study. Future studies with a broad range of patients with thyroid eye disease addressing these limitations might improve our understanding of the results of the intraoperative relaxed muscle positioning technique.

## Conclusion

Although surgery for the patients with TED related strabismus is challenging in terms of achieving excellent success, in this report we have shown that IRMP technique in which the extent of recession was determined by marking the natural place of the released tendon during the primary position might be a very successful option for correcting the strabismus. Despite limited number of patients, we have shown a dramatic increase in the QoL of the patients after the surgery by providing no diplopia in primary and reading gazes without prisms in 7 of 8 patients with only a single surgery. Further studies with larger sample sizes comparing a different surgical technique might provide us a better understanding of the results of this unique technique.

## Additional file

Additional file 1: **Table S1.** Graves Ophthalmopathy-Quality of Life Scores of the patients. This file includes the preoperative and postoperative visual functioning and appearance score of the patients. (DOCX 13 kb)

## Abbreviations

ET: Esotropia
GO-QoL: Graves Ophthalmopathy-QoL
IRMP: Intraoperative relaxed muscle positioning
IRR: Inferior rectus recession
MRR: Medial rectus recession
PD: Prism diopter
QoL: Quality of Life
TED: Thyroid Eye Disease
