# Supplementary material for: Intraoperative relaxed muscle positioning technique results in a tertiary Center for Thyroid Orbitopathy Related Strabismus
Source: BMC Ophthalmol. 2018 Nov 23;18:305. doi: 10.1186/s12886-018-0974-0 (PMC6251154; doi:10.1186/s12886-018-0974-0)
Supplement: Supplementary file 1 — Table S1. Graves Ophthalmopathy-Quality of Life Scores of the patients. This file includes the preoperative and postoperative visual functioning and appearance score of the patients. (DOCX 13 kb) [file 12886_2018_974_MOESM1_ESM.docx]

**Supplementary Table 1.** Graves Ophthalmopathy-Quality of Life Scores of the patients.

|  | **pre-VF** | **%** | **pre-AP** | **%** | **post-VF** | **%** | **post-AP** | **%** |
| --- | --- | --- | --- | --- | --- | --- | --- | --- |
| **1** | 8 | 0 | 9 | 6.3 | 24 | 100 | 23 | 93.8 |
| **2** | 8 | 0 | 9 | 6.3 | 24 | 100 | 23 | 93.8 |
| **3** | 8 | 0 | 8 | 0 | 14 | 37.5 | 14 | 37.5 |
| **4** | 8 | 0 | 8 | 0 | 24 | 100 | 23 | 93.8 |
| **5** | 9 | 6.25 | 8 | 0 | 24 | 100 | 24 | 100 |
| **6** | 9 | 6.25 | 8 | 0 | 24 | 100 | 24 | 100 |
| **7** | 9 | 6.25 | 8 | 0 | 24 | 100 | 24 | 100 |
| **8** | 9 | 6.25 | 8 | 0 | 24 | 100 | 24 | 100 |

VF: Visual functioning score

AP: Appearance score

pre: preoperative

post: postoperative
